# A comparison of the Scottish Index of Multiple Deprivation (SIMD) 2004 with the 2009 + 1 SIMD: does choice of measure affect the interpretation of inequality in mortality?

**DOI:** 10.1186/1476-072X-13-27

**Published:** 2014-07-08

**Authors:** Kevin Ralston, Ruth Dundas, Alastair H Leyland

**Affiliations:** 1 University of Edinburgh, Edinburgh, UK; 2 MRC/CSO Social and Public Health Sciences Unit, University of Glasgow, Glasgow, UK

**Keywords:** Deprivation, Inequality, Mortality, Measurement, Scottish Index of Multiple Deprivation (SIMD)

## Abstract

**Background:**

There is a growing international literature assessing inequalities in health and mortality by area based measures. However, there are few works comparing measures available to inform research design. The analysis here seeks to begin to address this issue by assessing whether there are important differences in the relationship between deprivation and inequalities in mortality when measures that have been constructed at different time points are compared.

**Methods:**

We contrast whether the interpretation of inequalities in all-cause mortality between the years 2008-10 changes in Scotland if we apply the earliest (2004) and the 2009 + 1 releases of the Scottish Index of Multiple Deprivation (SIMD) to make this comparison. The 2004 release is based on data from 2001/2 and the 2009 + 1 release is based on data from 2008/9. The slope index of inequality (SII) and 1:10 ratio are used to summarise inequalities standardised by age/sex using population and mortality records.

**Results:**

The 1:10 ratio suggests some differences in the magnitude of inequalities measured using SIMD at different time points. However, the SII shows much closer correspondence.

**Conclusions:**

Overall the findings show that substantive conclusions in relation to inequalities in all-cause mortality are little changed by the updated measure. This information is beneficial to researchers as the most recent measures are not always available. This adds to the body of literature showing stability in inequalities in health and mortality by geographical deprivation over time.

## Background

There is an expanding international literature assessing inequalities in health and mortality by area based measures [1-3]. The measurement of material deprivation is synonymous with the measurement of poverty. In general indices of multiple deprivation are intended to capture the multidimensionality of the concept of deprivation and the poverty it signifies [4]. This stems from an academic and policy interest in understanding the complex nature of poverty and its association with negative health and social outcomes with a view to reducing these [5]. The UK has a strong history in constructing these types of tools. These include the Townsend Scale [6] Carstairs Index [7], the Index of Multiple Deprivation – IMD [8] and the Scottish equivalent, the Scottish Index of Multiple Deprivation – SIMD [9].

Deprivation measures such as Townsend and Carstairs are based on Census data. In the UK there has been a move away from this toward the use of more routinely collected administrative data (e.g. SIMD, IMD). The primary motivation in this has been to produce measures that can be regularly updated and which may therefore be of more use to policy makers [10]. In New Zealand the 5 year census cycle means measures such as NZDep [11] have likewise been updated several times. The South African Index of Multiple Deprivation (SAIMD) has also been updated following its inception [12]. This leads to the question of whether an ongoing study should update a measure if it becomes available during an analysis period. It may be that the policy imperative to have regular updates to aid resource allocation is not always matched in academic research where substantive interests or populations of interest could be less open to the influence of short term fluctuations.

Deprivation indexes have a diversity of research and policy uses. The primary policy roles of indices may be in the allocation of resources, assessing need and evaluating policy effectiveness [13]. The New Zealand ELSI (Economic and Living Standards Index) was established with the aim of capturing deprivation but also to describe living standards for the population as a whole [14]. Examples of substantive research areas where the ELSI has been applied include the study of health and health inequalities [15,16], standards of living [17], ageing and retirement [18,19] and socio-economic position [20]. The Bavarian Index of Multiple Deprivation (BIMD) was developed specifically in reference to measuring regional differences in health outcomes [21]. Early work using the BIMD has therefore focussed on health and mortality [22-26]. Indeed, measures of area based deprivation are regularly used within public health research. Picket and Pearl [26] review the literature and show that area based measures make a contribution to explaining health outcomes when individual measures are controlled. A study of measures of socio-economic position in New Zealand came to similar conclusions [20]. This suggests the importance of robust tools to take account of the contextual in policy and research [27].

Various countries have now established measures of multiple deprivation [4]. For example indexes are available in Australia [28], Japan [29] and New Zealand [11,14]. A lot of work has been conducted within Europe for example, Layte et al. [30] has generated a composite index from European Community Household Panel data. A recent German study has developed the BIMD drawing directly on method developed in the UK [21] and the SAIMD [31] has done similarly.

There is also some research available which compares measures. In an early example of this Morris and Carstairs [32] compared five area based measures and their relationship to health outcomes at a postcode sector level in Scotland. Keriger et al. [33] undertook comparisons of Carstairs, Towsnend, composite variables and a composite index operationalised at different geographical levels, and applied to all-cause and cause-specific mortality in the USA. They found measures of economic deprivation to be the most robust at capturing socio-economic gradients in mortality, and that a larger geographical resolution was less reliable. Adams and White [34] compared the effectiveness of IMD 2004 in examining health inequalities with and without the health domain, finding little difference between either forms of the index. Bertin *et al*. [35] analyse a region of France to assess whether Carstairs, Townsend, Harvard or Rey measures can be uses legitimately across urban and rural contexts in relation to health needs. Of these measures they find Carstairs to be the most relevant in both urban and rural settings.

Within the UK there is a literature which highlights the consistency of geographical patterns of deprivation, even over long periods of time [36,37]. For example, Dorling *et al*. [36] compared the relationship between deprivation in London in 1896 and 1991 and showed that a measure of area based deprivation constructed with historical data correlated with patterns of deprivation a century later (r = 0.73). Indeed they showed that the historical deprivation measure had more explanatory power in predicting mortality from stroke and stomach cancer than a contemporary equivalent. Gregory [37] similarly found that a measure constructed from historical Census and national statistics data for 614 districts of England and Wales in the early 1900s related strongly to mortality at the end of the 20^th^ Century.

The contribution of this research is to assess whether there are important differences in the relationship between deprivation and mortality when SIMD measures that have been constructed at different time points and from different data are used. This is an original contribution to the discourse as we compare an index which is regularly updated. This also expands on previous work showing the historical consistency of areal deprivation in the measurement of health outcomes and feeds into the growing international use of measures of area deprivation to examine health and health inequalities. How consistent we can expect results to be when using measures constructed differently is potentially of wide interest. We therefore compare how the assessment of inequalities in mortality according to area-based deprivation between the years 2008-10 changes in Scotland if we apply deprivation measures from different times. We use the earliest, 2004, and the 2009 + 1 releases of SIMD to make this comparison.

It has been the intention from the conception of the SIMD that regular updates be applied [38]. It has received major updates in 2006 and 2009 when all seven domains were updated. Each domain consists of several indicators compiled from data that are able to be updated on a regular basis. At each major update the data used for the indicators may arise from differing data sources. This is due to the nature of the indicators themselves changing due to policy changes. The data points from which the original index (SIMD 2004) is constructed are at times 7 years apart from the data used for the SIMD 2009 (see Additional file 1: Table S1). For example, the SIMD 2004 income domain (used in these analyses) is constructed using data from the Department of Work and Pensions from as early as 2001 [9]. However, the 2009 release of SIMD was created using benefits and tax data from 2008 [39]. This was further updated using data from 2009 in a subsequent release, SIMD 2009 + 1 (see Additional file 1: Table S1). The differing data sources could render analysis based upon an older SIMD out of date and official advice is to use the index closest to the year for which data to be analysed is drawn [40]. Moreover, some of the small areas from which SIMD is constructed are subject to high levels of population change [41] opening the possibility of change in deprivation scores.

## Results and discussion

**Figure 1:**
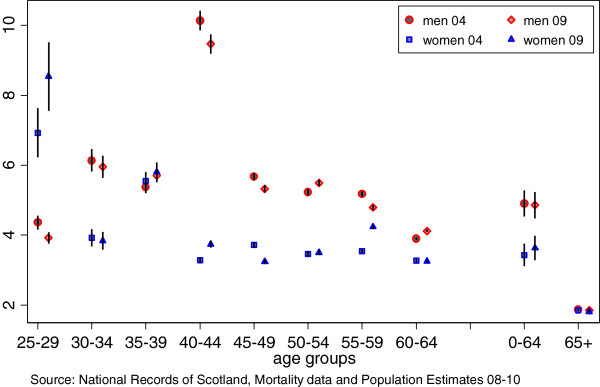
1:10 Ratio and 95% confidence intervals comparing SIMD 04 and 09+1 by 5 year and 0-64 and 65+ age groups.

**Figure 2:**
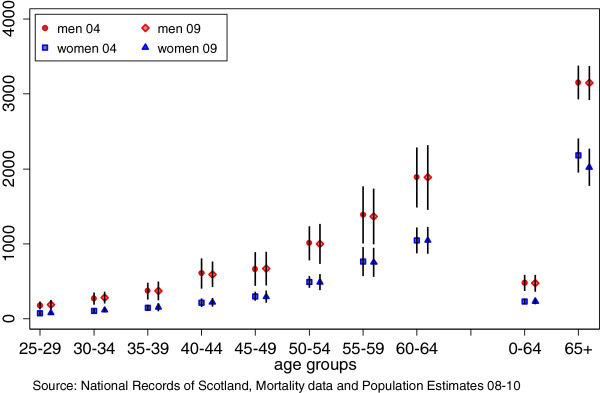
Slope Index of Inequality and 95% confidence intervals comparing SIMD 04 and 09+1 by 5 year and 0-64 and 65+ age groups.

SIMD 2004 and SIMD 2009 + 1 are strongly correlated (r = 0.955). The frequency ratio shows some differences according to the year on which the SIMD was based (Figure 1). For example, the largest inequalities are seen for men aged 40-44 but the ratio is estimated to be 10.1 (CI 9.9-10.4) using SIMD 2004 and 9.5 (CI 9.2-9.7) using SIMD 2009 + 1. The direction of the effect is not consistent; whilst the frequency ratios for men aged 50-54 and 55-59 were both 5.2 using SIMD 2004 (CI 5.1-5.3, 5.1-5.3), the corresponding figures using SIMD 2009 + 1 were 5.5 (CI 5.4-5.6) and 4.8 (CI 4.7-4.9). The SII shows much closer agreement over the two time points and the confidence intervals overlap for the age groups (Figure 2). It is possible to find instances of relatively large differences between the mortality rates estimated for a particular age group in one deprivation decile – such as for men aged 35-39 in decile 7, with a mortality rate of 140 (CI 130-149) and 95 (CI 87-102) per 100,000 using SIMD 2004 and 2009 respectively (Additional file 2: Table S2). The overall pattern, however, is one of fairly high correspondence between the two SIMD measures.

SIMD is officially sanctioned by the Scottish Government and is regularly updated. Advice is to employ the SIMD measure which is closest to the year in which the data to be analysed are drawn and dependent upon what analysis is to be undertaken [40]. However, we have shown that using the updated measure makes marginal differences in the examination of all-cause mortality or inequalities. Indeed, the fact that the difference appears larger using the frequency ratio than the SII suggests that the choice of measure of inequalities may be more important than the version of the deprivation index used. Further research is required to check the consistency of SIMD when applied to other outcomes. This is also needed in the case of other regularly updated measures (e.g. NZDep, SAIMD and IMD).

### Strengths and limitations

We only considered all-cause mortality as an outcome. This has previously been criticised as being an insensitive measure by Frank and Haw [42] who show that socioeconomic position potentially has a misleading effect when comparing measures at different time points. We are also unable to draw conclusions regarding whether the use of measures derived from different time points may have a substantive impact on the many other uses for which a deprivation measure such as SIMD is used (for example the analysis of other health and non-health-related outcomes). The strengths of this research are that we uniquely compare recent measures of deprivation applied to small area geographies at a national level to examine all-cause mortality using data on the entire population. Our findings echo previous research comparing indices and measures constructed on geographies of deprivation [36,37]. These studies show high correlation between outcomes, such as mortality, when comparing recent with historical geographies of deprivation. Our work adds to this discourse by comparing results when using two constructions of the SIMD applied to all-cause mortality at one time. Also, SIMD is based on much smaller geographical areas than, for example, the ward areas considered by Dorling [36].^a^It might be expected that larger areas have a more consistent deprivation profile over time than smaller areas.

## Conclusion

These findings show that substantive conclusions in relation to inequalities in all-cause mortality, at these small levels of aggregation, are little changed by the use of a contemporary measure of deprivation compared to a measure based on data from 10 years previous to the mortality data. Differences are more pronounced for the frequency ratio than the SII. This reflects the fact that the SII uses data for the entire population whilst the frequency ratio, using data from just the two most extreme groups, is more sensitive to minor changes in the indices. This information is important and useful for researchers who may have to choose which version of SIMD to use for an analysis or where there is only one SIMD measure. The results add to the body literature showing the consistency of geographically measured inequality in health and mortality over time and also feeds into the growing international discourse in the use of area based measures in researching health and health inequalities.

## Methods

SIMD is created using seven different domains to score geographies on their relative deprivation. These are employment, income, health, education, access, crime and housing. The overall SIMD rank is a weighted sum of the 7 domains; the income domain contributes 28% to the overall score [43]. The SIMD is operationalized at the level of datazone (mean population ~780, n = 6505); the income domain is used to group datazones into deciles (weighted by population) and, in general terms, this domain provides a count of the population living in households in receipt of means tested benefits and income support. The income domain is used here in isolation when studying mortality since the overall index incorporates the health domain which includes a measure of mortality [34]. The income domain is highly correlated with the overall SIMD (r = 0.98). Additional file 1: Table S1 provides a breakdown of the data used in the construction of the SIMD indices for the income domain at different time points. The original income domain of the SIMD 2004, although released in 2004, was created using data from 2001 and 2002. Data from the Department of Work and Pensions and the Inland Revenue, were used; indicators included data on Disability Tax Credit and Working Families Tax Credit data from 2002. This was replaced with Working and Child Tax Credit data for the 2006 update. However, SIMD 2009 reintroduced Disability Tax Credit and Working Families Tax Credit data and the updated version of SIMD 2009, released in 2009, uses data from 2008. The SIMD income domain was further updated with the release of SIMD 2009 + 1 using data from 2009. Inequalities in mortality were assessed using the income domain of SIMD 2004 and SIMD 2009 + 1. Thus comparing the measure using data drawn from as early as 2001 (SIMD 2004) with data from 2009 (the updated SIMD 2009 + 1, see Additional file 1: Table S1).

Geocoded mortality and population data were available for all deaths by age and sex for years 2008-10. Age standardised all-cause mortality rates were calculated in five year age groups for men and women between the ages of 25 and 64, together with summary measures for those aged 0-64 and 65+ (see Additional file 2: Table S2). Inequalities in mortality rates, according to the year of the SIMD, were examined by sex, age and deprivation deciles. The frequency ratio (R1:10) was calculated to measure relative inequality between the most and least deprived deciles. This is the ratio of the relative frequency of mortality among the most deprive decile (10) to the least deprived (1) [44], and was estimated with corresponding confidence intervals [45]. The slope index of inequality (SII) per 100,000 population was calculated to summarize absolute inequalities [46]. This estimates the absolute difference in rates between the most and least deprived extremes of the distribution, taking into account all groups in the population. SAS version 9.2 was used to calculate the mortality rates and the SII, the figures were produced using Stata version 11.

## Endnote

^a^Ward areas have a mean population of around 5500, whereas the SIMD datazones have an average population size 780.

## Abbreviations

BIMD: Bavarian index of multiple deprivation; CI: Confidence interval; ELSI: Economic and living standards index; IMD: Index of multiple deprivation; NZDep: New Zealand small-area index of relative socio-economic deprivation; SAIMD: South African index of multiple deprivation; SII: Slope index of inequality; SIMD: Scottish index of multiple deprivation.

## Competing interests

The authors declare that they have no competing interests.

## Authors’ contributions

AL conceived of the research question and design and contributed in final edits. KR conducted the main body of the research and drafted the paper including producing graphics and tables. RD contributed statistical expertise including practical input in standardising mortality rates and constructing the SII measure, RD also contributed to editing the paper. All authors read and approved the final manuscript.

## Authors’ information

Professor Alastair Leyland is Associate Director and head of the programme “Measuring Health, Variations in Health and the Determinants of Health” at the MRC/CSO Social and Public Health Sciences Unit, University of Glasgow.

Ruth Dundas is a Senior Investigator Scientist at MRC/CSO Social and Public Health Sciences Unit, University of Glasgow.

Dr Kevin Ralston is a Research Fellow at the University of Edinburgh.

## Supplementary Material

Additional file 1: Table S1 Data used in the construction of the Scottish Index of Multiple Deprivation income domain, at different time points. Click here for file

Additional file 2: Table S2 Mortality rates by five year age and 0-64 and 65+ age groups. Click here for file
