# Supplementary material for: A comparison of the Scottish Index of Multiple Deprivation (SIMD) 2004 with the 2009 + 1 SIMD: does choice of measure affect the interpretation of inequality in mortality?
Source: Int J Health Geogr. 2014 Jul 8;13:27. doi: 10.1186/1476-072X-13-27 (PMC4105786; doi:10.1186/1476-072X-13-27)
Supplement: Additional file 1: Table S1 — Data used in the construction of the Scottish Index of Multiple Deprivation income domain, at different time points. [file 1476-072X-13-27-S1.docx]

Table S1

| **Data used in the construction of the Scottish Index of Multiple Deprivation income domain, at different time points** | | |
| --- | --- | --- |
| *2004* | *2009* | *2009+1* |
| Adults in Income Support households (DWP April 2002) | Number of Adults (aged 16-59) receiving Income (Department for Work and Pensions (DWP) August 2008) | From 2008 Employment and Support Allowance (ESA) replaced Incapacity Benefit and Income Support paid on the grounds of incapacity for new claimants. People receiving income-based ESA are included in the revised 2009+1 income domain. The dependent benefit data used in the SIMD 2009+1 income domain is updated from 2008 to 2009 data. |
| - | Number of Adults (aged 60 plus) receiving Guaranteed Pension Credit (DWP August 2008) |  |
| Children in Income Support  households (DWP April 2002) | Number of Children (aged 0-15) dependent on a recipient of Income Support (DWP August 2008) |  |
| Adults in Income Based Job Seekers  Allowance households (DWP August  2001) | Number of Adults receiving (all) Job Seekers Allowance (DWP August 2008) |  |
| Children in Income Based Job Seekers  Allowance households (DWP August  2001) | Number of Children (aged 0-15) dependent on a recipient of Job Seekers Allowance (all) (DWP August 2008) |  |
| Adults in Working Families Tax Credit  Households below a low income  threshold (DWP / IR April 2002) | Number of Adults and Children in Tax Credit Families on low incomes (HMRC 2008) |  |
| Children in Working Families Tax  Credit Households below a low income  threshold (DWP / IR April 2002) |  |  |
| Adults in Disability Tax Credit  households below a low income  threshold (DWP / IR April 2002) |  |  |
| Children in Disability Tax Credit  households below a low income  threshold (DWP / IR April 2002) |  |  |
| ***Source:*** *Scottish Executive (2004) Scottish Government (2009) Scottish Government (2011)* | | |
