# Supplementary material for: A comparison of the Scottish Index of Multiple Deprivation (SIMD) 2004 with the 2009 + 1 SIMD: does choice of measure affect the interpretation of inequality in mortality?
Source: Int J Health Geogr. 2014 Jul 8;13:27. doi: 10.1186/1476-072X-13-27 (PMC4105786; doi:10.1186/1476-072X-13-27)
Supplement: Additional file 2: Table S2 — Mortality rates by five year age and 0-64 and 65+ age groups. [file 1476-072X-13-27-S2.docx]

Table S2

**Mortality rates by five year age and 0-64 and 65+ age groups**

| *All cause mortality 2008-10 by deciles* ***men*** *and age groups* ***simd04*** | | | | | | | | |  |  |
| --- | --- | --- | --- | --- | --- | --- | --- | --- | --- | --- |
| deciles | **25-29** | **30-34** | **35-39** | **40-44** | **45-49** | **50-54** | **55-59** | **60-64** | **0-64** | **65+** |
| **1** | 243 | 351 | 469 | 740 | 869 | 1216 | 1882 | 2546 | 609 | 6693 |
| **2** | 164 | 246 | 343 | 470 | 642 | 931 | 1292 | 2084 | 456 | 6110 |
| **3** | 118 | 177 | 247 | 329 | 461 | 804 | 1155 | 1793 | 376 | 5783 |
| **4** | 127 | 173 | 234 | 345 | 383 | 674 | 979 | 1466 | 322 | 5489 |
| **5** | 135 | 113 | 162 | 248 | 345 | 563 | 870 | 1330 | 285 | 5228 |
| **6** | 117 | 136 | 140 | 214 | 280 | 487 | 775 | 1218 | 249 | 4912 |
| **7** | 69 | 93 | 140 | 145 | 259 | 348 | 582 | 1019 | 201 | 4549 |
| **8** | 67 | 71 | 107 | 116 | 195 | 327 | 565 | 865 | 175 | 4190 |
| **9** | 45 | 86 | 82 | 102 | 200 | 260 | 468 | 758 | 149 | 3949 |
| **10** | 56 | 57 | 87 | 73 | 153 | 233 | 364 | 653 | 124 | 3585 |
| *Ratio 1:10* | *4.3* | *6.2* | *5.4* | *10.1* | *5.7* | *5.2* | *5.2* | *3.9* | *4.9* | *1.7* |
| SII | *180* | *271* | *372* | *605* | *665* | *1008* | *1389* | *1887* | *480* | *3154* |
|  | | | | | | | | | | |

| *All cause mortality 2008-10 by deciles* ***men*** *and age groups* ***simd09+1*** | | | | | | | | |  |  |
| --- | --- | --- | --- | --- | --- | --- | --- | --- | --- | --- |
| deciles | **25-29** | **30-34** | **35-39** | **40-44** | **45-49** | **50-54** | **55-59** | **60-64** | **0-64** | **65+** |
| **1** | 256 | 352 | 475 | 691 | 859 | 1263 | 1818 | 2576 | 609 | 6572 |
| **2** | 171 | 231 | 351 | 490 | 656 | 898 | 1362 | 2060 | 455 | 6229 |
| **3** | 117 | 231 | 241 | 342 | 459 | 739 | 1039 | 1629 | 358 | 5735 |
| **4** | 113 | 157 | 199 | 326 | 361 | 678 | 966 | 1523 | 324 | 5373 |
| **5** | 127 | 126 | 202 | 271 | 320 | 508 | 894 | 1312 | 280 | 5143 |
| **6** | 113 | 103 | 135 | 170 | 297 | 440 | 760 | 1204 | 237 | 4854 |
| **7** | 87 | 93 | 95 | 147 | 261 | 401 | 524 | 950 | 196 | 4420 |
| **8** | 54 | 71 | 133 | 129 | 186 | 344 | 584 | 838 | 176 | 4341 |
| **9** | 36 | 80 | 89 | 98 | 174 | 242 | 455 | 815 | 147 | 3924 |
| **10** | 65 | 59 | 83 | 73 | 161 | 230 | 379 | 625 | 125 | 3562 |
| *Ratio 1:10* | *3.9* | *6.0* | *5.7* | *9.5* | *5.3* | *5.5* | *4.8* | *4.1* | *4.9* | *1.8* |
| *SII* | *188* | *283* | *372* | *593* | *672* | *1001* | *1367* | *1887* | *476* | *3146* |

| *All cause mortality 2008-10 by deciles* ***women*** *and age groups* ***simd04*** | | | | | | | | |  |  |
| --- | --- | --- | --- | --- | --- | --- | --- | --- | --- | --- |
| deciles | **25-29** | **30-34** | **35-39** | **40-44** | **45-49** | **50-54** | **55-59** | **60-64** | **0-64** | **65+** |
| **1** | 91 | 141 | 195 | 277 | 387 | 622 | 1041 | 1472 | 308 | 4887 |
| **2** | 71 | 110 | 159 | 242 | 340 | 552 | 871 | 1290 | 267 | 4416 |
| **3** | 34 | 93 | 120 | 216 | 282 | 482 | 694 | 1082 | 219 | 4241 |
| **4** | 37 | 67 | 107 | 139 | 257 | 398 | 596 | 1025 | 192 | 4045 |
| **5** | 37 | 63 | 72 | 135 | 186 | 373 | 529 | 919 | 173 | 3797 |
| **6** | 49 | 58 | 90 | 126 | 194 | 324 | 489 | 732 | 156 | 3771 |
| **7** | 22 | 36 | 73 | 106 | 171 | 246 | 367 | 669 | 125 | 3487 |
| **8** | 18 | 44 | 68 | 95 | 140 | 221 | 349 | 613 | 115 | 3202 |
| **9** | 15 | 39 | 60 | 78 | 126 | 207 | 320 | 564 | 105 | 2917 |
| **10** | 13 | 36 | 35 | 84 | 104 | 180 | 294 | 450 | 90 | 2648 |
| *Ratio1:10* | *7* | *3.9* | *5.6* | *3.3* | *3.7* | *3.5* | *3.5* | *3.3* | *3.4* | *1.8* |
| *SII* | *73* | *108* | *149* | *217* | *302* | *492* | *764* | *1048* | *2332* | *2179* |

| *All cause mortality 2008-10 by deciles* ***women*** *and age groups* ***simd09+1*** | | | | | | | | |  |  |
| --- | --- | --- | --- | --- | --- | --- | --- | --- | --- | --- |
| deciles | **25-29** | **30-34** | **35-39** | **40-44** | **45-49** | **50-54** | **55-59** | **60-64** | **0-64** | **65+** |
| **1** | 91 | 147 | 215 | 290 | 379 | 647 | 1064 | 1426 | 314 | 4704 |
| **2** | 72 | 114 | 135 | 228 | 375 | 529 | 791 | 1304 | 256 | 4428 |
| **3** | 41 | 76 | 125 | 197 | 252 | 428 | 646 | 1162 | 215 | 4206 |
| **4** | 43 | 93 | 106 | 150 | 219 | 454 | 643 | 904 | 196 | 3887 |
| **5** | 27 | 64 | 69 | 158 | 214 | 346 | 527 | 867 | 167 | 3861 |
| **6** | 36 | 51 | 80 | 107 | 179 | 291 | 434 | 729 | 141 | 3647 |
| **7** | 37 | 31 | 76 | 105 | 158 | 244 | 399 | 693 | 133 | 3444 |
| **8** | 14 | 41 | 88 | 82 | 126 | 213 | 359 | 601 | 113 | 3348 |
| **9** | 12 | 30 | 38 | 87 | 137 | 214 | 345 | 562 | 105 | 3064 |
| **10** | 11 | 38 | 37 | 78 | 117 | 185 | 251 | 439 | 86 | 2595 |
| *Ratio 1:10* | *8.3* | *3.9* | *5.8* | *3.7* | *3.2* | *3.5* | *4.2* | *3.2* | *3.7* | *1.8* |
| *SII* | *79* | *117* | *154* | *222* | *294* | *490* | *754* | *1047* | *233* | *2021* |

***Source:*** *National Records of Scotland death registration data 2008-2010, midyear population estimates 2008-2010*
